# Real-Time Visualization of HIV-1 GAG Trafficking in Infected Macrophages

**DOI:** 10.1371/journal.ppat.1000015

**Published:** 2008-03-07

**Authors:** Karine Gousset, Sherimay D. Ablan, Lori V. Coren, Akira Ono, Ferri Soheilian, Kunio Nagashima, David E. Ott, Eric O. Freed

**Affiliations:** 1 Virus-Cell Interaction Section, HIV Drug Resistance Program, National Cancer Institute, Frederick, Maryland, United States of America; 2 AIDS Vaccine Program, SAIC-Frederick, Inc., National Cancer Institute, Frederick, Maryland, United States of America; 3 Department of Microbiology and Immunology, University of Michigan Medical School, Ann Arbor, Michigan, United States of America; 4 Image Analysis Laboratory, Advanced Technology Program, SAIC-Frederick, National Cancer Institute at Frederick, Frederick, Maryland, United States of America; Northwestern University, United States of America

## Abstract

HIV-1 particle production is driven by the Gag precursor protein Pr55^Gag^. Despite significant progress in defining both the viral and cellular determinants of HIV-1 assembly and release, the trafficking pathway used by Gag to reach its site of assembly in the infected cell remains to be elucidated. The Gag trafficking itinerary in primary monocyte-derived macrophages is especially poorly understood. To define the site of assembly and characterize the Gag trafficking pathway in this physiologically relevant cell type, we have made use of the biarsenical-tetracysteine system. A small tetracysteine tag was introduced near the C-terminus of the matrix domain of Gag. The insertion of the tag at this position did not interfere with Gag trafficking, virus assembly or release, particle infectivity, or the kinetics of virus replication. By using this *in vivo* detection system to visualize Gag trafficking in living macrophages, Gag was observed to accumulate both at the plasma membrane and in an apparently internal compartment that bears markers characteristic of late endosomes or multivesicular bodies. Significantly, the internal Gag rapidly translocated to the junction between the infected macrophages and uninfected T cells following macrophage/T-cell synapse formation. These data indicate that a population of Gag in infected macrophages remains sequestered internally and is presented to uninfected target cells at a virological synapse.

## Author Summary

The viral Gag protein is both necessary and sufficient for the assembly of a new generation of virus particles. There has been a significant amount of debate in recent years regarding the site in the cell at which HIV-1 assembly takes place. Of particular interest has been the site of assembly in macrophages, a cell type that serves as an important target for HIV-1 infection *in vivo*. In this study, we examine the site of Gag localization and virus assembly in primary human macrophages in living cells by using biarsenical dyes that become fluorescent when they bind a small target sequence introduced into HIV-1 Gag. We observe Gag localization both at the plasma membrane and in an apparently internal compartment that bears markers characteristic of multivesicular bodies (MVBs). Significantly, when infected macrophages are cocultured with uninfected T cells, the apparently internal Gag moves rapidly to the contact site, or synapse, between the macrophage and the T cell. These findings support the hypothesis that infected macrophages sequester assembled HIV-1 particles in an internal compartment and that these particles move to synapses where cell–cell transmission can occur.

## Introduction

The human immunodeficiency virus type 1 (HIV-1) Gag polyprotein precursor, Pr55^Gag^, plays an essential role in virus assembly and release. Its expression alone is able to generate virus-like particles (VLPs) [1],[2]. All four domains of Pr55^Gag^–matrix (MA), capsid (CA), nucleocapsid (NC) and p6–play important roles in particle assembly and release [1],[3]. The MA domain regulates the association of Gag with the host cell plasma membrane (PM); this membrane-binding activity is provided primarily by a myristic acid moiety covalently attached to the N-terminus of MA and a highly basic patch of amino acid residues that interacts with acidic phospholipids, including phosphatidylinositol-(4,5)-bisphosphate [PI4,5)P_2_] on the inner leaflet of the PM [4],[5],[6],[7]. CA and NC promote Gag-Gag interactions during assembly [8], in part through the ability of NC to interact with nucleic acid [2],[9]. Finally, the p6 domain of Gag stimulates virus release by interacting with components of the cellular endosomal sorting machinery [10],[11],[12].

Although significant progress has been made in elucidating the viral and cellular factors necessary for Gag membrane binding, Gag multimerization, and virus release, the subcellular location of HIV-1 assembly has been the subject of controversy and the itinerary of Gag trafficking to the site of assembly remains to be defined. Mutational studies have shown that the viral determinants for Gag targeting to the PM reside in the MA domain of Gag. A large deletion in MA redirects HIV-1 assembly to the endoplasmic reticulum [13],[14], whereas point mutations, particularly in the highly basic domain of MA, shift the site of assembly from the PM to internal compartments [15],[16],[17] defined as late endosomes or multivesicular bodies (MVBs) [18].

HIV-1 was long assumed to follow the classically defined “C-type” pathway in which Gag assembly and release take place at the PM [2]. This dogma was challenged by a number of studies suggesting that HIV-1 assembly takes place in an endosomal compartment and that particle release from the infected cell follows the “exosomal” pathway in which virus-containing endosomes fuse with the PM to release their contents [19],[20],[21],[22],[23],[24]. This endosomal model was then subsequently contested by several studies showing PM-based HIV-1 assembly and release [25],[26],[27],[28],[29]. The nature of the HIV-1 assembly site in primary monocyte-derived macrophages (MDMs) has been a matter of particular interest [30]. Early electron microscopy (EM) observations in HIV-1-infected MDMs revealed an abundance of virions assembling and budding into intracellular vacuoles [31],[32]. In later studies, it was observed that the virus-containing internal compartments in MDMs bore markers characteristic of late endosomes or MVBs; e.g., major histocompatibility complex II (MHC II) and tetraspanins CD63, CD81, and CD82 [18],[33],[34]. Furthermore, virions derived from MDMs packaged late endosome/MVB markers, suggesting that these virions originated from a late endosomal compartment [33],[35],[36]. In an intriguing refinement of the model that HIV-1 assembles in MVBs in primary macrophages, it was demonstrated that at least some of the virus-positive, “intracellular” structures in MDMs were actually connected to the PM. These apparently internal structures may therefore represent PM invaginations that are positive for tetraspanin markers [37],[38]. Elucidating the virus assembly pathway in primary MDMs is highly significant since this cell type represents one of the major targets for HIV-1 infection *in vivo* [39].

One of the difficulties in evaluating previous studies focused on defining the Gag assembly/release pathway in MDMs is the absence of live-cell imaging data in this cell type that allow the trafficking of Gag to be visualized in real time. To this end, we developed a system for visualizing in living cells the localization and trafficking of Gag expressed in the context of a fully infectious and replication-competent HIV-1 molecular clone. We used the biarsenical-tetracysteine labeling method first described by Tsien and colleagues [40],[41],[42]. This system is based on the insertion of a small tetracysteine (TC) motif into a protein of interest. Cells expressing the TC-tagged protein are treated with a membrane-permeable biarsenical dye [e.g., green (FlAsH) or red (ReAsH)] that fluoresces upon binding to the TC tag. The advantages of this method are that the TC tag is very small and that labeling occurs immediately upon binding of the dye to the TC tag. Recently, this system was used to label Gag expressed from non-infectious clones in HeLa, Mel Juso and Jurkat T cells [23],[29]. We introduced the TC tag near the C-terminus of the MA domain of Gag in the context of the full-length infectious HIV-1 molecular clone pNL4-3. Insertion of the TC tag had no significant effect on HIV-1 Gag function. By using VSV-G-pseudotyped viruses, we were able to infect and follow Gag trafficking in primary MDMs. Our data indicate that in MDMs Gag accumulates both at the PM and in an apparently internal MVB-like compartment. Although we obtained no evidence for constitutive movement of the internal Gag to the PM, or for internalization of the PM-localized Gag to apparently internal structures, we observed rapid relocation of the internal population of Gag to the site of cell-cell contact following addition of susceptible T cells to the infected macrophage cultures. These findings support a model whereby newly assembled virus particles are sequestered in infected macrophages and then efficiently presented to susceptible target cells following synapse formation.

## Results

### Introduction of a TC tag near the C-terminus of the MA domain of HIV-1 Gag does not disrupt virus replication, assembly and release, or Gag trafficking

**Figure 1:**
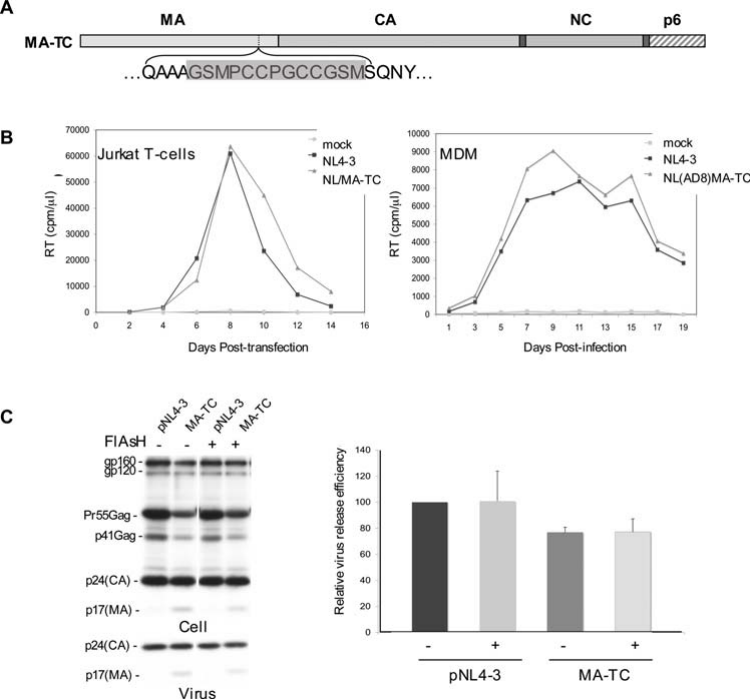
The MA-TC tag does not affect Gag function. (A) Schematic diagram of HIV-1 Gag indicating the position of TC tag insertion. The amino acid sequence of the TC tag is shaded. (B) Replication kinetics of WT HIV-1 vs. the MA-TC derivative in the Jurkat T-cell line and primary MDM. Jurkat and MDM experiments were performed with pNL4-3 and pNL(AD8) molecular clones, respectively. Media were obtained every two days for RT analysis. (C) Virus release efficiency of WT vs. MA-TC. HeLa cells were transfected with WT pNL4-3 or pNL4-3/MA-TC plasmids. Transfected cells were labeled with FlAsH or DMSO (control) for 5 min 24–48 hrs posttransfection and were washed for 20 min with 300 µM EDT/PBS. The cells were then metabolically labeled with [^35^S]Met/Cys for 2 hrs. Released virions were pelleted by ultracentrifugation, and both cell and virus lysates were immunoprecipitated with HIV-Ig and subjected to SDS-PAGE. Bands were quantified using a phosphorimager. +/− SD, n = 3.

The MA domain of HIV-1 Gag performs several important functions in virus assembly and release [1]; however, deletion of a number of C-terminal residues (amino acids 116-128) [43], or the insertion of a Myc or green fluorescent protein (GFP) tag near the C-terminus of MA [44] does not block virus assembly and release, suggesting that the C-terminus of MA is relatively insensitive to mutation. Thus, to facilitate the study of HIV-1 Gag trafficking, we deleted codons 121–128 of MA and inserted a TC tag in the full-length molecular clone pNL4-3 to generate pNL4-3/MA-TC (Fig. 1A).

To determine the effects of the TC tag on virus replication, we transfected the Jurkat T-cell line with WT pNL4-3 or with pNL4-3/MA-TC and monitored virus replication over time by measuring the levels of reverse transcriptase (RT) activity in the medium (Fig. 1B). We observed that replication of NL4-3/MA-TC was comparable to that of WT in Jurkat T-cells. To test the replication of MA-TC in primary MDMs, the MA-TC tag was introduced into the macrophage-tropic pNL4-3 derivative pNL(AD8) [45],[46]. Virus stocks were prepared and used to infect MDMs. As indicated in Fig. 1B, the NL(AD8)/MA-TC virus replicated with kinetics indistinguishable from those of WT NL(AD8) in this physiologically relevant primary cell type.

The ability of MA-TC virus to replicate efficiently in both T-cell lines and primary MDMs suggested that the insertion of the TC tag near the C-terminus of MA does not affect HIV-1 assembly or release. To test this directly, we transfected HeLa cells with WT pNL4-3 or pNL4-3/MA-TC. One day posttransfection, the cells were labeled for 5 minutes with or without FlAsH, washed with ethanedithiol (EDT), and metabolically labeled for 2–3 hrs with [^35^S]Met/Cys. Cell and virion lysates were prepared, immunoprecipitated with anti-HIV immunoglobulin (HIV-Ig), subjected to SDS-PAGE, and bands were quantitated by phosphorimager analysis (Figure 1C) (see Materials and Methods). The results indicated that insertion of the MA-TC tag had no significant effect on virus particle production and that the FlAsH dye caused no measurable disruption of HIV-1 particle production. We note that insertion of the TC tag in MA resulted in increased labeling of the MA protein with [^35^S]Met/Cys due to the additional Cys residues (Fig. 1C). We also compared the single-cycle infectivity of WT and MA-TC Gag in the TZM-bl indicator cell line [47] and observed no effect of the MA-TC tag on virus infectivity (data not shown). Together, these data demonstrate that the MA-TC tag does not disrupt normal HIV-1 Gag function.

**Figure 2:**
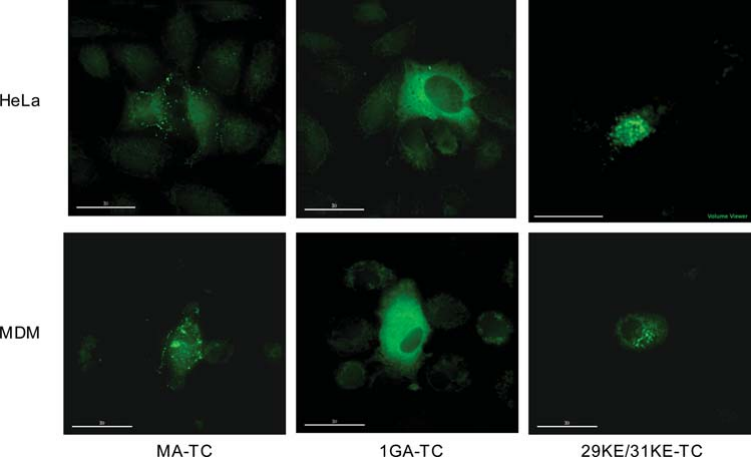
The MA-TC tag does not affect Gag localization. HeLa cells (top panel) were transfected with pNL4-3/MA-TC or 1GA or 29KE/31KE derivatives. Cells were labeled with FlAsH for 5 min at 37°C, washed, fixed in 3.7% formaldehyde, and examined microscopically. MDMs (bottom panel) were infected with VSV-G- pseudotyped virus stocks that transduced NL4-3/MA-TC or 1GA or 29KE/31KE derivatives (see Materials and Methods). Infected cells were labeled and fixed as described above. Scale bars = 30 µm. For a 3D z-series of the 29KE/31KE-TC mutant in MDM, see Video S1.

We previously reported a number of mutations within the MA domain of Gag that alter normal HIV-1 Gag trafficking and localization [15],[16],[18]. For example, mutation of the site of Gag myristylation (1GA; [15]) results in a diffuse cytosolic Gag localization. Mutations in the MA highly basic domain (e.g., 29KE/31KE) retarget Gag to MVBs [16],[18]. To validate further the TC labeling approach, we sought to confirm that the effect of these mutations on Gag localization in the context of otherwise WT Gag would be recapitulated in the context of MA-TC Gag. We introduced the 1GA and 29KE/31KE MA mutations into MA-TC and analyzed Gag localization within cells using a rapid FlAsH labeling method. Transfected HeLa cells were labeled for 5 min with FlAsH and washed for 20 min in EDT. Cells were then fixed and either mounted or processed further for antibody labeling. Similar to our previous results obtained with antibody labeling [16],[48], MA-TC Gag was found primarily in a punctate pattern at the cell surface. In contrast, MA-TC/1GA was diffusely localized throughout the cytosol and MA-TC/29KE/31KE was found in internal compartments (Fig. 2, top). We previously observed that the internal compartment to which 29KE/31KE localizes in HeLa cells is positive for the MVB marker CD63 [18]. To verify that this was also the case in the context of MA-TC Gag, we examined the colocalization of the 29KE/31KE-TC mutant, labeled with ReAsH, with CD63 in transfected HeLa cells. We observed nearly complete colocalization between 29KE/31KE Gag and CD63 (Fig. S1). These results confirm the biochemical experiments indicating that the addition of the TC tag had no effect on Gag trafficking, assembly, or release in HeLa cells.

Since our goal was to visualize Gag trafficking in physiologically relevant primary cells, we analyzed the localization pattern of MA-TC Gag in infected MDMs. Cells were infected with VSV-G-pseudotyped virus stocks obtained from transfected 293T cells. Infected MDMs were then labeled with FlAsH and fixed 24 to 72 hours post-infection. Infection efficiencies, as determined by Gag staining, typically ranged between 2 and 10%. MA-TC Gag localized both to the PM and to an apparently internal compartment (Fig. 2, bottom). In agreement with our previous results obtained by antibody labeling [16],[18],[49], the localization of MA-TC-derived 1GA and 29KE/31KE Gag in MDMs was similar to that observed in HeLa cells: 1GA-TC was diffusely distributed throughout the cytoplasm, and 29KE/31KE-TC was almost exclusively found in apparently internal compartments (Fig. 2, bottom). To provide a clearer visualization of the internal localization of 29KE/31KE-TC Gag in MDM, we obtained a z-series reconstruction by using the Maximum Intensity Projection mode from the image processing software OsiriX (Video S1). The results presented in Fig. 2 demonstrate that introduction of the TC tag near the C-terminus of MA (MA-TC) allows HIV-1 Gag to be readily visualized in infected primary MDMs at early time points postinfection.

### In MDM, Gag localizes to both the PM and to an internal, tetraspanin-positive compartment

**Figure 3 ppat-1000015-g003:**
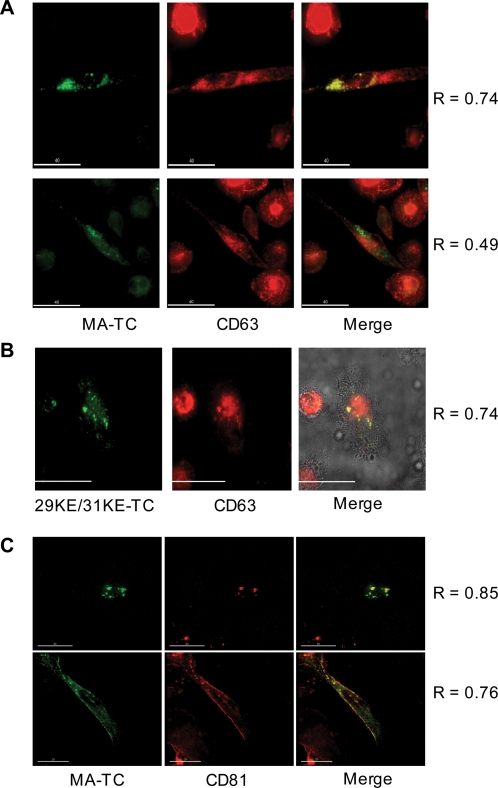
Gag in MDMs colocalizes with tetraspanins at the cell surface and in apparently internal compartments. MDMs infected with VSV-G-pseudotyped NL4-3/MA-TC (A and C) or NL4-3/29KE/31KE-TC (B) were labeled with FlAsH (green) 20 hours post-infection, fixed, and further labeled with antibody against CD63 (red) (A and B) or CD81 (red) (C). The merged images of Gag-TC and CD63/CD81 are shown on the right, with yellow indicating colocalization between Gag and CD63/CD81. R = Pearson coefficient of correlation. Scale bars: panel A, 40 µm; panel B, 30 µm; panel C; 30 µm top, 20 µm bottom.

We and others have previously reported that the apparently internal vesicles to which HIV-1 Gag localizes in MDMs bear tetraspanin markers, suggesting that they are MVBs or MVB-like structures [18],[33],[34]. To define the site of Gag localization in MDMs at early time points postinfection using TC-tagged Gag, we infected MDMs and examined the localization of Gag and tetraspanins (CD63 and CD81) at 20 hrs postinfection. As previously observed with fully WT Gag [18], MA-TC Gag displayed a localization pattern that partially overlapped with that of CD63 (Fig. 3A). The colocalization pattern in these cells was very heterogeneous, with some cells displaying a high degree of Gag/CD63 colocalization (Fig. 3A, top panels) and other cells showing a lower level of colocalization (Fig. 3A, lower panels). 29KE/31KE-TC Gag also overlapped with a subset of CD63 in infected MDMs (Fig. 3B). Both MA-TC (Fig. 3C) and 29KE/31KE-TC Gag (data not shown) showed much more extensive colocalization with CD81 than with CD63. We note that some cells displayed a high level of Gag and CD81 costaining at the PM (Fig. 3C, lower panel), consistent with HIV-1 assembly occurring in tetraspanin-enriched microdomains at the cell surface [19],[50]. To quantitatively compare the degree of Gag/CD63 vs. Gag/CD81 colocalization in MDMs, we measured the Pearson correlation coefficient (R) values (see Materials and Methods) for these two sets of colocalizing proteins in a total of 75 cells. The results confirmed the higher degree of Gag/CD81 compared to Gag/CD63 colocalization (Fig. S2). We observed that 71% of cells displayed a Gag/CD63 R-value of <0.6, whereas 91% of cells showed a Gag/CD81 R-value of >0.6 (Fig. S2).

**Figure 4:**
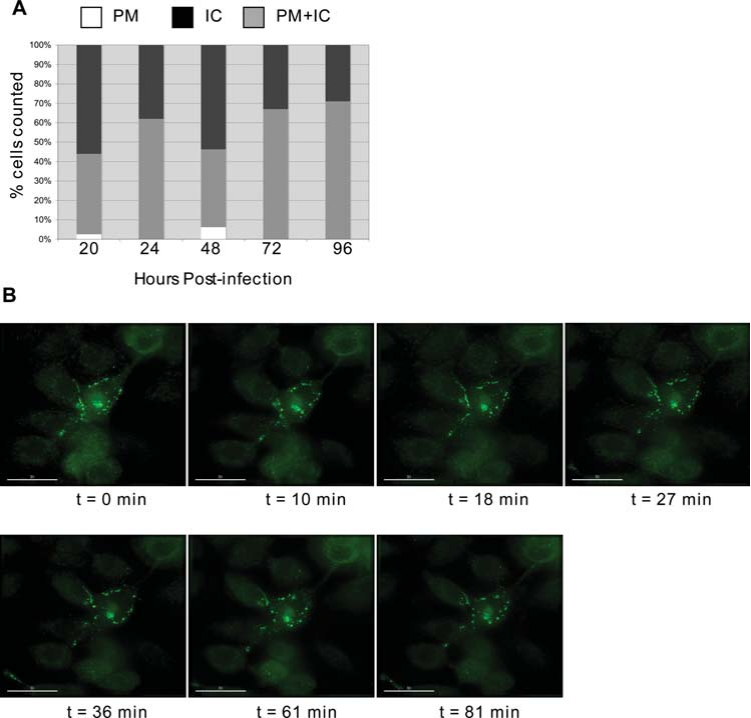
Gag localization in MDMs remains stable over time. (A) MDMs were labeled with FlAsH 20, 24, 48, 72, and 96 hrs after infection with VSV-G-pseudotyped NL4-3/MA-TC. At each time point, between 25 and 65 cells were categorized as having PM, intracellular (IC), or both PM+intracellular (PM+IC) Gag localization. (B) Live-cell analysis. MDMs infected with VSV-G-pseudotyped NL4-3/MA-TC virus were labeled with FlAsH 48 hrs postinfection, and immediately examined microscopically on the stage in a closed chamber (37°C/5% CO_2_). Time (t) represents time in min after FlAsH labeling. Scale bar = 30 µm.

As indicated in Fig. 3, WT Gag was localized both to an internal tetraspanin-positive compartment and to the PM in infected MDMs. Very few cells showed exclusively PM staining; instead, the vast majority of cells showed either an internal localization or both PM and internal staining. To determine whether the distribution changed over time, we classified cells as displaying uniquely PM, intracellular, or both PM and intracellular Gag localization at 20, 24, 48, 72, and 96 hrs postinfection. The percentage of cells within these three categories remained essentially unchanged over time (Fig. 4A).

To visualize Gag movement in living MDMs, cells were infected with MA-TC virions pseudotyped with VSV-G and were labeled for 5 min with FlAsH 24 to 72 hrs post-infection. After washing, labeled MDMs were immediately placed in a microscope chamber (37°C/5% CO_2_) and imaged over time. Interestingly, no clear movement of Gag between PM and apparently intracellular compartments was observed during the time course (Fig. 4B); i.e., no obvious internalization of Gag from the PM was visualized, nor was there clear movement of internal Gag puncta to the PM. These results suggest that Gag can assemble both at the PM and in internal compartments in infected MDMs. As stated in the Materials and Methods, prior to 20 hrs post-infection we were not able to definitively distinguish between specific Gag staining and the diffuse, low-level background.

### Gag and CD81 accumulate at sites of cell-to-cell contact

**Figure 5:**
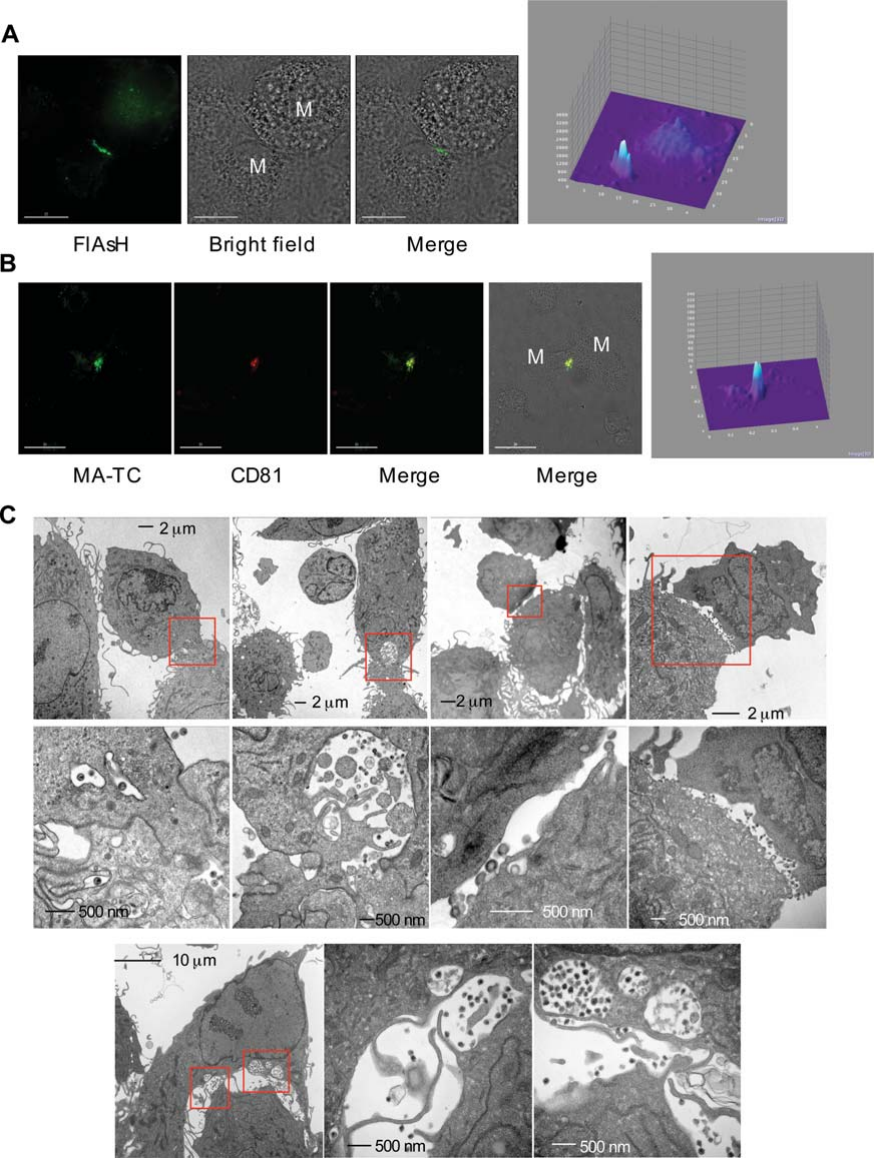
Gag accumulates at the MDM/MDM synapse. Cells were infected with VSV-G-pseudotyped NL4-3/MA-TC virus, labeled with FlAsH 24–48 hrs post-infection, and fixed in 3.7% formaldehyde. (A) Gag accumulates at the synapse between MDMs. Individual macrophages are labeled “M”. Scale bar = 15 µm. (B) After FlAsH labeling, cells were fixed and stained with anti-CD81 antibody. The extensive overlap between Gag and CD81 at the MDM/MDM synapse is visualized as yellow in the merged panels. Scale bar = 30 µm. Far-right panels in (A and B) provide quantification of the Gag signals. The Surface Plot analyzing tool from the ImageJ software was used to obtain a three-dimensional graph of the pixel intensities in grayscale. The x- and y- axes represent the length of the region analyzed in pixels, and the z-axis represents the pixel intensity of the Gag signal. (C) EM analysis of VLPs at the synapse. Infected MDMs were fixed with 2% glutaraldehyde and processed for EM. Fully assembled VLPs can be visualized at the MDM/MDM junctions. Red boxes represent regions that are enlarged in the adjacent panels.

During the course of our analyses, we frequently observed concentrated Gag staining at the contact sites formed between infected and uninfected MDMs (Fig. 5A). 3D z-stack reconstructions illustrating this phenomenon are presented in Figs. S3A and Video S2). These Gag-enriched cell-cell junctions also displayed a high degree of staining for the tetraspanin markers CD81 and CD82 (Fig. 5B and data not shown). Analogous junctions have been reported to form between HIV-1-treated dendritic cells and T-cells; because these junctions bear markers (e.g., tetraspanins and adhesion molecules) found at immunological synapses [51],[52],[53] they have been named “infectious” or “virological” synapses [54],[55],[56],[57],[58]. A concentration of budding and released virions was also observed in the vicinity of cell-cell contact sites by transmission electron microscopy (EM) (Fig. 5C). To quantify the localization of Gag at the synapse observed in our EM analysis, we counted the number of virus particles and budding structures at synapse vs. non-synapse regions of the plasma membrane. More than 60 cells were scored for this analysis. The results indicated a markedly (5-6-fold) higher density of particles and budding events at synapse vs. non-synapse regions of the cell surface, consistent with the immunofluorescence data presented above.

**Figure 6:**
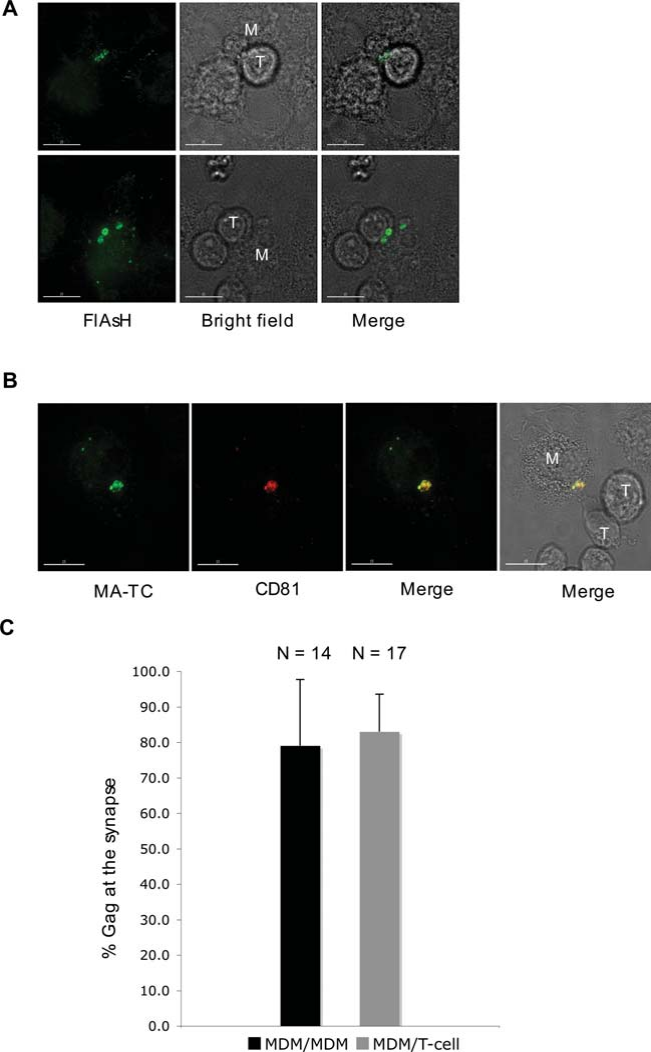
Gag accumulates at the MDM/T-cell synapse. (A) Infected MDMs were labeled with FlAsH and Jurkat cells were added to the cultures. One to two hours after addition of the T-cells, the co-cultures were fixed with 3.7% formaldehyde and imaged or (B) stained with anti-CD81 antibody. In panel B, Gag/CD81 colocalization is indicated as yellow in merge. M = MDMs; T = Jurkat T-cells. Scale bars in panels A and B = 15 µm. (C) Quantification of Gag concentration at the synapse. The microscopy images were opened in ImageJ and a plot profile was obtained. A column average plot was generated, in which the x-axis represents the horizontal distance through the image and the z-axis the vertically averaged pixel intensity. The % Gag at the synapse = (pixel intensity of Gag signal at synapse)/(total intensity of Gag in the cell+synapse)×100. N values indicate the number of cells analyzed in each data set. For more information on how the Gag quantification was performed, see Fig. S3A.

To extend the analysis of Gag concentration at the cell-cell synapse to include junctions formed between infected MDMs and uninfected T-cells, we performed the following analysis: infected MDMs were labeled with FlAsH and then incubated at 37°C for 2 hours with Jurkat T-cells. The cells were then fixed and, when necessary, labeled with anti-CD81 antibodies. Gag was frequently detected at the synapses between infected MDMs and uninfected Jurkat T-cells (Fig. 6A). 3D z-stack reconstructions are provided in Videos S3 and S4. Furthermore, as we observed for MDM/MDM junctions, MDM/T-cell synapses also displayed a high degree of colocalization between Gag and tetraspanin markers (Fig. 6B). We also observed that Gag concentrated at synapses formed between infected MDMs and primary T cells (data not shown). Overall, these data show that HIV-1 Gag, along with CD81, are recruited to the synapses formed between infected macrophages and uninfected macrophages or T-cells. To quantify the concentration of Gag at the synapse, we used the ImageJ software to determine the pixel intensity for Gag staining at the MDM/MDM and MDM/T-cell synapses compared to the overall pixel intensity in each infected cell. The results confirmed a high degree of Gag concentration at cell-cell junctions, with approximately 80% of the total Gag signal localized to the synapse (Figs. 6C, S3A).

### Movement of Gag to the synapse in MDM is not Env-dependent but is disrupted by mutations in MA

**Figure 7:**
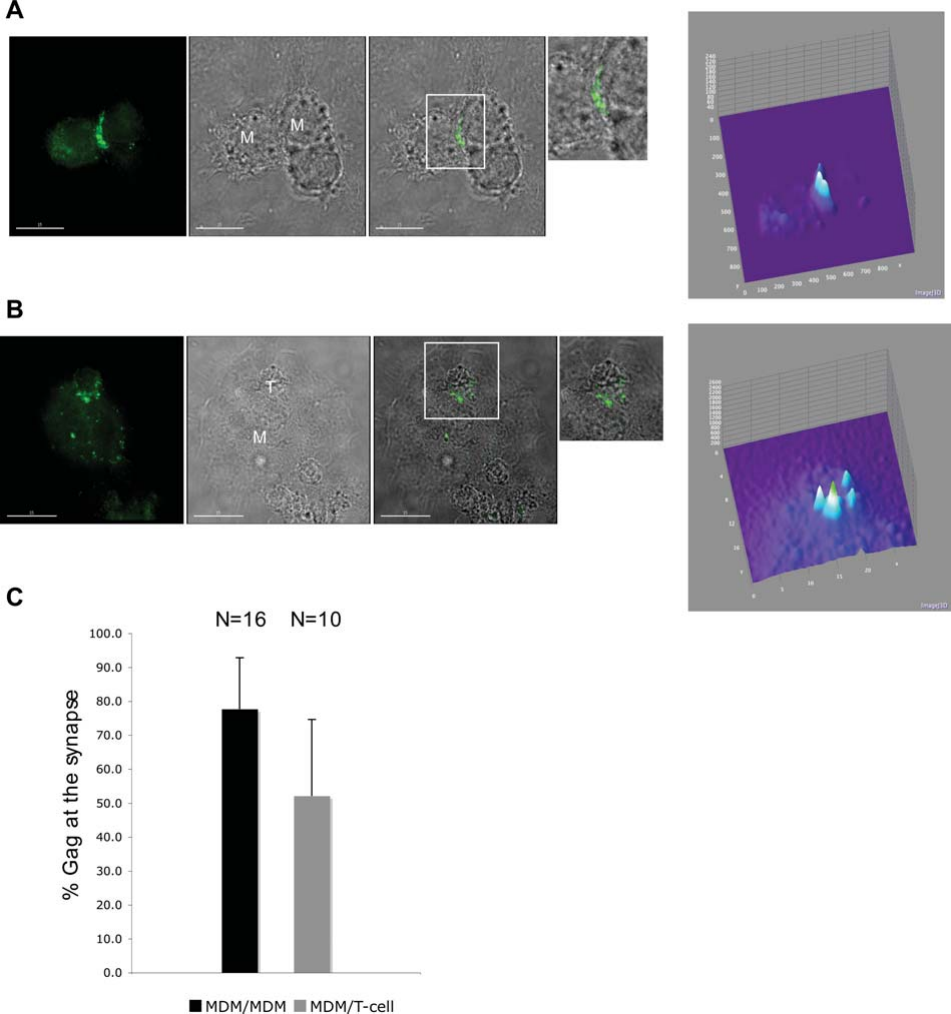
Gag recruitment to the synapse in MDMs is Env-independent. (A) MDMs were infected with VSV-G-pseudotyped NL4-3/KFS/MA-TC virus (which is defective for HIV-1 Env expression) and were labeled with FlAsH 24–48 hrs postinfection. Boxes indicate regions enlarged on the right. Scale bars = 15 µm. (B) MDMs were infected as in (A), and Jurkat T cells were added after FlAsH labeling. Far-right panels in (A) and (B) provide quantification of the Gag signals, determined as indicated in the Fig. 5 legend. Scale bars = 15 µm. (C) Quantification of Gag concentration at the synapse, determined as described in the Fig. 6C legend. For more information on how the Gag quantification was performed, see Fig. S3B.

To analyze further the process of Gag recruitment to the synapse in infected MDMs, we determined whether Gag was recruited to cell-cell junctions in the context of proviral clones carrying additional mutations. We first examined the localization of Gag in the absence of Env expression by using the Env(-) MA-TC mutant KFS/MA-TC. Examining a possible role for Env in Gag recruitment to the MDM synapse was of interest as it has been reported that Env is required for synapse formation between infected and uninfected T cells [59] and also plays a role in the formation of filopodial bridges that can facilitate transfer of retroviruses between cells [60]. In contrast to these prior findings in non-monocytic cell types, we observed that Gag was efficiently localized to both MDM/MDM and MDM/T-cell synapses in the absence of Env expression (Fig. 7A and B). This concentration of Gag to the synapse was quantified as described above, confirming the high degree of localization of Gag to the cell-cell junction independent of Env expression (Fig. 7C, S3B). 3D z-stack reconstructions are provided in Video S5. The data indicated no statistically significant difference between Gag concentration at the MDM/T-cell vs, MDM/MDM synapse, or in the presence or absence of Env expression (compare Figs. 6C and 7C).

**Figure 8:**
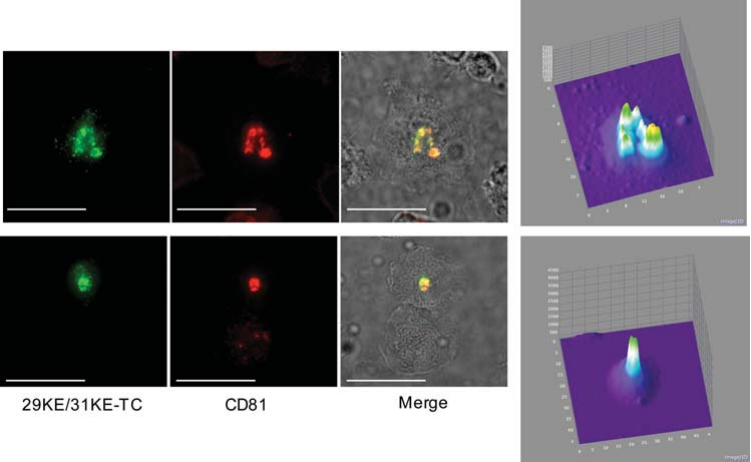
Gag recruitment to the synapse in MDMs is blocked by the 29KE/31KE MA mutations. MDMs were infected with VSV-G-pseudotyped NL4-3/29KE/31KE-TC virus, labeled with FlAsH 24–48 hrs postinfection, fixed, and stained with anti-CD81 antibody. Gag/CD81 colocalization is indicated as yellow in merge. Scale bars = 30 µm. Far-right panels show distribution of Gag signal; plots were obtained as described in the Fig. 5 legend. Note the centrally located (non-synapse) concentration of Gag in these cells expressing the 29KE/31KE MA mutant.

We previously reported that mutations in the highly basic domain of MA (e.g., 29KE/31KE) redirect Gag to MVBs [18]. Here, we observed that in MDMs the 29KE/31KE-TC mutant displayed nearly complete localization to an apparently internal compartment that stained positive for CD63 and CD81 (Figs. 2 and 3 and data not shown). In contrast, MA-TC Gag displayed a mix of PM and internal staining (e.g., Fig. 2–4). It was therefore of interest to examine whether 29KE/31KE-TC Gag could redistribute from its normally internal site of localization to the cell surface upon synapse formation. Interestingly, we observed that in contrast to MA-TC Gag, 29KE/31KE-TC Gag did not relocalize to either MDM/MDM (Fig. 8; Video S6) or MDM-T-cell (data not shown) synapse. Instead, in cells expressing 29KE/31KE-TC Gag, both Gag and CD81 remained deep within the infected cell (Fig. 8). In four independent experiments with 29KE/31KE-TC, Gag accumulation was never observed at the synapse. These data suggest the possibility that the apparently internal compartments to which WT and 29KE/31KE Gag localize are distinct.

### Real-time visualization of Gag movement to the infectious synapse in living MDM

**Figure 9:**
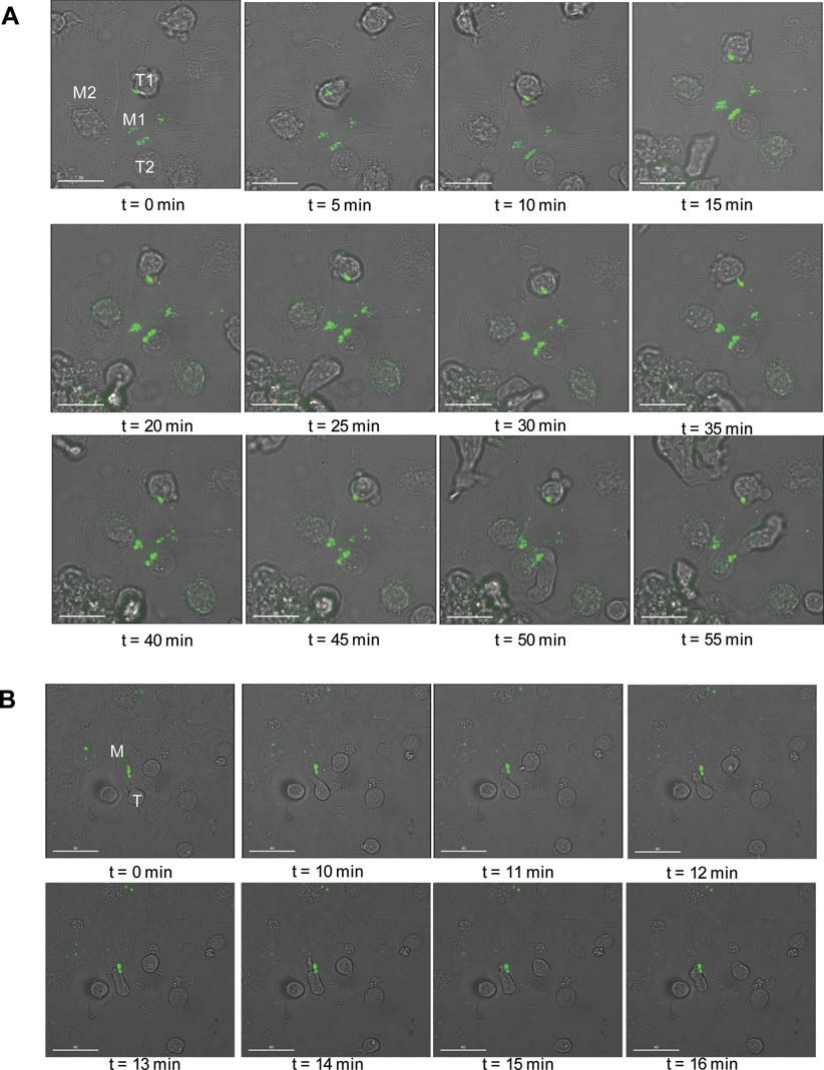
Real-time trafficking of Gag to the synapse in infected MDMs. (A) MDMs infected with VSV-G-pseudotyped NL4-3/MA-TC virus were labeled with FlAsH 48 hrs postinfection. Jurkat T-cells were added to the FlAsH-labeled cultures and incubated on the microscope stage in a closed chamber (37°C /5% CO_2_). Time t = 0 min is 40 min (gallery A) or 70 min (gallery B) after addition of Jurkat cells. M1 = infected MDM; M2 = non-infected MDM; T1 and T2 are Jurkat T-cells. Scale bars = 20 µm in A and 40 µm in B. For a movie of Gag movement to the synapse, derived from the experiment presented in Fig. 9A, see Video S7.

The data presented above using fixed infected cells and EM techniques demonstrate the accumulation of Gag and virus particles at the junction between infected MDMs and uninfected MDMs or T-cells. To visualize the movement of Gag to the cell-cell contact site, we used FlAsH labeling and live-cell imaging in infected MDMs. For these experiments, infected MDMs were labeled with FlAsH for 5 minutes 24 to 72 hours post-infection, washed, and imaged over time. When visualizing MDM/T-cell junctions, Jurkat T-cells were added to the infected cells post-FlAsH labeling and imaged under the same conditions. After addition of the Jurkat cells, incubation periods of approximately 30–60 min were required for stable MDM/T-cell synapses to form. As mentioned above, we observed no clear evidence of movement of apparently internal Gag to the PM, or vice versa, in MDMs not actively engaged in cell-cell contact. Interestingly, however, upon addition of Jurkat T-cells to the infected MDM cultures, we observed rapid movement of apparently internal Gag to the MDM/T-cell synapse. The infected macrophage (“M1”) in Fig. 9A is surrounded by uninfected macrophages (e.g., “M2”) and Jurkat T-cells (“T1” and “T2”). In this particular time course, 40 min after adding Jurkat T cells to the MDMs (t = 0 min), Gag has already accumulated at the contact site between M1 and T1. Gag-containing compartments are also rapidly recruited to the site of M1/T2 contact. Movement of other Gag-containing compartments toward the site of MDM-MDM (M1/M2) contact can be observed starting at time t = 25 min and is complete at t = 40 min. These data demonstrate that Gag present in internal compartments can be rapidly redistributed to the site of contact with uninfected cells. After its movement to the MDM/MDM synapse, Gag can be seen moving along the surface of macrophage M2 (e.g., at 45 and 50 min). A movie of Gag movement to the synapse can be viewed at Video S7. Intriguingly, we frequently observed an apparent preference for MDM/T-cell synapse formation at sites close to high levels of Gag concentration (Fig. 9B). In this gallery, time t = 0 represents cells 90 min post-FlAsH labeling and 70 min after addition of T-cells. One of the surrounding T-cells (“T “) at time t = 0 min extends on top of the infected MDM toward the site of Gag accumulation. This resulted in the movement and attachment of the T-cell with the infected MDM near the site of Gag accumulation.

## Discussion

Most studies that have examined HIV-1 Gag trafficking have used non-infectious constructs in which codon-optimized Gag is fused to fluorescent proteins such as green or red fluorescent protein (GFP or RFP). Although these studies provided important insights, disadvantages of using GFP and its derivatives in protein trafficking analyses include the large size of the fluorescent protein and the fact that achieving their fluorescent state requires time-dependent chromophore maturation [61],[62]. We have also observed that Gag expressed from some codon-optimized constructs assembles relatively inefficiently and forms perinuclear cytosolic aggregates not typically observed with WT Gag (unpublished results). In this study, we describe the application of the biarsenical labeling system to visualize HIV-1 Gag trafficking in primary MDMs. We show here that the TC tag that serves as the binding site for the biarsenical dye FlAsH is remarkably well tolerated with respect to preserving Gag function when introduced near the C-terminus of the MA domain. The MA-TC tagged Gag produces virus particles with WT efficiency, and these particles are fully infectious in both single-cycle assays and in spreading infections. The MA-TC Gag can be readily delivered to primary cells as a VSV-G pseudotype. Overall, this system provides a rapid and efficient method for observing WT Gag trafficking in living cells.

The biarsenical labeling system employed in this study allowed us to examine Gag localization after ∼20 hrs postinfection, the earliest time point at which Gag expression could be readily and consistently visualized. At this early time point, we observed a mix of PM and apparently internal Gag staining. Between 20 and 96 hrs postinfection, we did not observe a shift in the percentage of cells displaying PM, intracellular, or PM+intracellular staining (Fig. 4) nor did we observe a time-dependent accumulation of internal Gag. These results differ from those of a recent study in which intracellular Gag-GFP staining increased over time [27]. We note that the TC-tagged Gag in the current study is fully functional for particle assembly and release and produces infectious virions. Furthermore, our TC-tagged Gag is expressed in the context of a full-length molecular clone that encodes all the HIV-1 accessory proteins including Vpu. Indeed, elimination of Vpu expression led to a time-dependent accumulation of Gag in internal compartments (unpublished results), consistent with recent reports [26],[28]. In agreement with the study of Jouvenet et al. [27], we did not observe an effect on virus release of treating infected cells with U18666A, a drug that arrests endosome motility (unpublished results). This observation supports the hypothesis that release of HIV-1 in MDMs occurs from PM-assembled VLPs. Although the FlAsH method can be accompanied by high background staining, we observed that this problem is largely mitigated by using very brief labeling periods. We also observed that background staining in MDM is less evident in MDM than in HeLa cells.

The most significant finding in this study is the visualization of apparently internal Gag moving to the site of cell-cell contact after synapse formation with uninfected T cells. It therefore appears that the tetraspanin-rich, apparently internal compartment in which HIV-1 assembles in MDMs can serve as a storage compartment for rapid presentation of virus particles at cell-cell junctions. These findings have clear implications for HIV-1 transmission from MDMs. In this regard, it is interesting to note that the virions in these internal vesicles reportedly remain infectious for weeks postinfection [63] and that virus transmission between infected MDMs and T cells is extremely rapid [64]. In several respects, our observations with infected MDMs are similar to those made with dendritic cells treated with HIV-1. Binding of HIV-1 virions to dendritic cells can lead to transfer of virus to uninfected T-cells through the formation of a virological or infectious dendritic cell/T-cell synapse without the dendritic cell itself being productively infected [55],[56],[57],[58],[65],[66]. Virions bound to the dendritic cell are reportedly internalized into an internal compartment that is strongly positive for CD81 but only weakly positive for CD63. Synapse formation induces the redistribution of virus particles and CD81 to the site of cell-cell contact, presumably facilitating transfer of virus to the T cell. The internal virus-containing compartment in dendritic cells is weakly acidic, as also reported for the virus-positive compartment in MDMs [67]. Thus, it appears that HIV-1 has evolved to subvert a pathway in both MDMs and dendritic cells that allows infectious virus particles to be retained in an apparently internal compartment and then redistributed to the cell surface following infectious synapse formation. Transfer of HIV-1 between T cells also involves the formation of a synapse that bears tetraspanin markers [68]; however, there is currently no evidence for long-term retention of infectious virus particles within an internal compartment in T cells. Interestingly, whereas the generation of T-cell/T-cell infectious synapses [59] and formation of cell-cell filopodial bridges that allow intercellular transfer of virus particles [60] have been reported to require Env expression, we found no such Env dependence for Gag translocation to the synapse formed between MDMs and T cells (Fig. 7A).

We previously reported that mutations in the highly basic domain of MA (e.g. 29KE/31KE) induce a shift in Gag localization in HeLa cells and T cells from PM to MVBs [18]. In MDMs, both WT and 29KE/31KE Gag localize to an apparently internal, tetraspanin-positive compartment [18]. This finding is confirmed here (e.g., Fig. 2 and 8). Interestingly, while WT MA-TC Gag rapidly translocates to the MDM/T-cell junction after synapse formation, we did not observe significant movement of 29KE/31KE-TC Gag to the MDM/T-cell synapse (Fig. 8). These results imply that WT and 29KE/31KE Gag localize to distinct tetraspanin-positive compartments in MDMs. A possible interpretation of these observations is that 29KE/31KE Gag localizes to “true” MVBs which do not move to the synapse, whereas WT Gag localizes to a compartment that is apparently internal but is connected to the PM [37],[38]. It is this PM-connected tetraspanin-positive compartment that undergoes a shift in localization following synapse formation, thereby allowing virus particle movement to the site of cell-cell contact. Surprisingly, we frequently observed that T-cells made contact with regions of the MDM PM under which Gag was concentrated, and in many cases the T-cells formed pseudopodia to contact this site (e.g., Fig. 9B). These results imply that the T cell can “sense” regions of the PM that overlie the putative invaginations in which assembled virus particles are concentrated. These regions of the PM may be enriched in lipid rafts and/or tetraspanin-enriched microdomains. These observations are somewhat reminiscent of previous studies on the recruitment of uninfected T cells into infected cell syncytia [69]. A future challenge will be to characterize in greater detail the membrane composition at the site of MDM/T-cell contact and elucidate the signals that induce the movement of the newly assembled, internally sequestered virus particles to the infectious synapse.

## Materials and Methods

### Plasmids and preparation of virus stocks

Plasmids pNL4-3/MA-TC and pNL(AD8)/MA-TC were constructed as follows: for pNL4-3/MA-TC, nucleotides 1250–1273 (encoding DTGNNSQV Gag codons 121–128) were deleted in the MA-coding region of the full-length HIV-1 molecular clone pNL4-3 [70] and the TC tag GSMPCCPGCCGSM was inserted in its place using overlap-extension PCR [71]. The MDM-tropic pNL(AD8)/MA-TC clone was constructed by exchanging the EcoRI-XhoI fragment of pNL4-3/MA-TC with that from the CCR5-tropic clone pNL(AD8) [45]. Construction of molecular clones expressing pNL4-3 MA mutants 1GA and 29KE/31KE was described previously [15],[16]. The molecular clones pNL4-3/1GA-TC and pNL4-3/29KE/31/KE-TC were constructed by exchanging the BssHII-SphI fragments of pNL4-3/1GA or pNL4-3/29KE/31KE with the corresponding fragments from MA-TC. To construct the Env(-) pNL4-3 construct, pNL4-3/KFS-TC, we exchanged the EcoRI-XhoI fragment from the Env(-) molecular clone pNL4-3/KFS [72] with the corresponding fragment from pNL4-3/MA-TC. Finally, we constructed pNL4-3/Vpu(-)/MA-TC by replacing the BssHII-EcoRI fragment from Vpu-DEL-1 [73] (kindly provided by K. Strebel), with the corresponding fragment from pNL4-3/MA-TC. VSV-G-pseudotyped virus stocks were prepared by transfecting 293T cells with the Gag/Pol expression vector pCMVNLGagPolRRE [74], the VSV-G expression vector pHCMV-G [75], and the indicated HIV-1 molecular clones by using Lipofectamine 2000 (Invitrogen), according to the manufacturer's protocol.

### Cells, transfections, and infections

HeLa and Jurkat T cells were cultured as previously described [15]. MDMs were prepared by culturing elutriated monocytes [45] in RPMI-1640 medium, supplemented with 10% fetal bovine serum, for 5 to 7 days on ultra-low attachment plates (Costar). HeLa cells were transfected by using the calcium phosphate method, as previously described [15]. Jurkat T-cells were transfected by using the DEAE-dextran procedure as previously reported [15]. Infection of MDMs was performed as follows: MDMs were detached from the ultra-low attachment plates (Fisher Scientific, Pittsburgh, PA) and plated onto tissue culture dishes or microscope culture chambers Fisher Scientific, Pittsburgh, PA). Virus stocks, pseudotyped with VSV-G, were incubated with MDMs for 5-6 hours. 2×10^6^ counts/minute (cpm) of reverse transcriptase (RT) activity was used per well of 4-well Nunc chambers, 10^6^ RT cpm/well for 8-well Nunc-chambers, and 4×10^6^ RT cpm/well for 6-well plates.

### Virus replication and infectivity assays

Virus replication assays in the Jurkat T-cell line were performed as previously described [15]. Briefly, Jurkat cells were transfected in parallel with WT pNL4-3 or MA-TC using the DEAE-dextran method. Cells were split 1∶3 every two days and an aliquot of medium was reserved at each time point for RT assay [76]. MDMs in 6-well plates were infected with 2×10^6^ RT cpm/well with WT pNL(AD8) or MA-TC(AD8) virus stocks. Medium in the infected MDM cultures was changed every two days and an aliquot was reserved for RT activity. For single-cycle infectivity assays, 4×10^5^ HeLa-derived TZM-bl cells [47] (obtained from J. Kappes through the NIH AIDS Research and References Reagent Program) per well were infected with 2×10^5^ RT cpm virus stocks. Infection efficiency was determined by measuring luciferase activity 2 days post-infection, as described previously [77].

### Biarsenical labeling

Adherent cells cultured in Lab-Tek chamber slides (Nunc) or 6-well plates were labeled 24–72 hours posttransfection/infection. All labeling steps were performed at 37°C in the dark. The cells were washed twice with Opti-MEM I (Invitrogen, Carlsbad). For each experiment, biarsenical labeling solutions were freshly prepared immediately prior to use. Wash solutions of 300 µM and 100 µM 1,2-ethaneditiol (EDT) (Aldrich Chemical Company, Inc., Milwaukee) were prepared in phosphate-buffered saline (PBS) and 0.2 mM Lumio Green (FlAsH) was prepared in dimethyl sulfoxide (DMSO) (Sigma-Aldrich, Inc., St Louis). Before labeling, 2 µl of 1 mM EDT was mixed quickly with 4.7 µl of 0.2 mM FlAsH or Lumio Red (ReAsH) and immediately added to 400 µl Opti-MEM I. This solution was added to cells, which were incubated for 5 min at 37°C. After the 5 min biarsenical labeling, cells were washed with 300 µM EDT/PBS for 8 min and 100 µM EDT/PBS for 10 min at 37°C. Cells were then washed further 3X with PBS and either fixed with 3.7% formaldehyde prior to antibody labeling or incubated with Opti-MEM I for live cell imaging or addition of Jurkat T cells. The levels of background in MDMs and in Hela cells were greatly reduced with the addition of EDT in our labeling solutions. We also observed that keeping the biarsenical labeling time short (5 min) was enough to obtain a strong Gag signal, while limiting non-specific background staining. As early as 20 hrs post-infection, specific Gag staining could be readily detected. However, at earlier time points, the cytosolic Gag signals were too low to be clearly distinguishable from background fluorescence, and therefore no data were acquired before 20 h postinfection.

### Fluorescence microscopy and EM

For fluorescence microscopy, 24–48 hours post transfection/infection, cells were labeled using the biarsenical method and either fixed using 3.7% formaldehyde/PBS for 20 min or Jurkat T-cells were added to the MDMs (in Opti-MEM I) for 2 hours, then fixed with formaldehyde. The cells were then permeabilized with 0.1% Triton-X100/PBS and incubated with 0.1 M glycine for 10 min at room temperature to quench free aldehyde groups. The cells were then blocked with 3% bovine serum albumin (BSA)/PBS, incubated with either mouse monoclonal anti-CD63 (Santa Cruz Biotechnology) or mouse monoclonal anti-CD81 (BD Pharmingen) for 1 hr at room temperature, washed and incubated with Alexa-594 or 488-conjugated anti-mouse IgG (Invitrogen) for 30 min at room temperature. The cells were then washed and mounted with Aqua Poly Mount (Polysciences Inc., Warrington, PA). For live cell imaging, the labeled cells were imaged in a temperature-controlled chamber (37°C/ 5% CO_2_) in Opti-MEM I. For both fixed and live-cell microscopy, the cells were imaged using an Olympus 1X-71 inverted deconvolution microscope and analyzed with Delta Vision software (Applied Precision Inc., Seattle, WA). To quantify the degree of relative colocalization, we obtained the Pearson correlation coefficient (R) values, which are standard measures of colocalization [78]. The R values were calculated using the softWORx colocalization module which generates a “colocalized” image from two channels. A scatter plot of the two intensities on a pixel-by-pixel basis is then plotted and the R value is calculated by dividing the covariances of each channel by the product of their standard deviations. For EM, infected cells were fixed and processed as previously described [15].

### Metabolic labeling and radioimmunoprecipitation analysis

Metabolic radiolabeling, preparation of cell and viral lysates, and immunoprecipitation assays were performed as previously described [15]. Briefly, transfected HeLa cells, or infected MDMs, labeled with the biarsenical dyes or DMSO (control) were metabolically labeled with [^35^S] Met/Cys for 2 hours, 24-48 hours posttransfection/infection, and released virions were pelleted by ultracentrifugation. Cell and virus lysates were immunoprecipitated with HIV immunoglobulin (HIV-Ig), obtained from NABI and the National Heart Blood and Lung Institute through the NIH AIDS Research and Reference Reagent Program. Immunoprecipitates were subjected to SDS-PAGE followed by fluorography. Quantitative analysis of the bands visualized by radioimmunoprecipitation was performed using a Bio-rad phosphorimager.

## Supporting Information

Figure S1 Colocalization of 29KE/31KE-TC Gag with CD63 in HeLa Cells. Cells transfected with pNL4-3/29KE/31KE-TC were labeled for 5 min with ReAsH (Gag) for 5 min at 37 °C, washed, fixed in 3.7% formaldehyde and then stained with anti-CD63 Ab (CD63). Lower panels show the overlay between the Gag and CD63 staining. Scale bars = 15 µm.
(4.20 MB TIF)

Figure S2 Quantification of Gag/CD63 and Gag/CD81 Colocalization. MDMs infected with VSV-G-pseudotyped were analyzed for Gag, CD63 and CD81 localization as described in the Fig. 3 legend. Pearson coefficient (R) values were obtained for a total of 75 cells and were plotted on a scale of 0 to 1 (x-axis). The y-axis indicates the number of cells scored within each R-value range.
(2.57 MB TIF)

Figure S3 Sample Images Used to Quantify the Localization of Gag at the Synapse. Illustrates method used for data presented in Fig. 6C (panel A) and Fig. 7C (panel B). The microscopy images were opened in ImageJ and a plot profile was obtained. A column average plot was generated, in which the x-axis represents the horizontal distance through the image and the y-axis the vertically averaged pixel intensity. The % Gag at the synapse was calculated as: (pixel intensity of Gag signal at synapse)/(total intensity of Gag in the cell+synapse)×100. N values indicate the number of cells analyzed in each data set. Scale bar = 10 µm.
(3.67 MB TIF)

Video S1 Analysis of 29KE/31KE-TC Gag localization in MDM in 3D. The z-series reconstructions were obtained by using the Maximum Intensity Projection mode from the image processing software OsiriX. The data correspond to the MDM 29KE/31KE-TC Gag panel in Fig. 2.
(0.18 MB MOV)

Video S2 Analysis of Gag localization in 3D. The z-series reconstructions were obtained by using the Maximum Intensity Projection mode from the image processing software OsiriX. The image in this figure corresponds to Fig. 5A.
(0.41 MB MOV)

Video S3 Analysis of Gag localization in 3D. The z-series reconstructions were obtained by using the Maximum Intensity Projection mode from the image processing software OsiriX. The image in this figure corresponds to Fig. 6A, bottom.
(0.38 MB MOV)

Video S4 Analysis of Gag localization in 3D. The z-series reconstructions were obtained by using the Maximum Intensity Projection mode from the image processing software OsiriX. The image in this figure corresponds to Fig. 6A, top.
(0.16 MB MOV)

Video S5 Analysis of Gag localization in 3D. The z-series reconstructions were obtained by using the Maximum Intensity Projection mode from the image processing software OsiriX. The image in this figure corresponds to Fig. 7A.
(0.30 MB MOV)

Video S6 Analysis of Gag localization in 3D. The z-series reconstructions were obtained by using the Maximum Intensity Projection mode from the image processing software OsiriX. The image in this figure corresponds to Fig. 8, top.
(0.13 MB MOV)

Video S7 Movie of Gag movement to the MDM/MDM and MDM/T-cell synapse. This experiment corresponds to the gallery presented in Fig. 9A.
(8.86 MB MOV)
